# A Tribute to Ricardo Gonzalez a Short Story Behind This Name

**DOI:** 10.3389/fped.2021.710379

**Published:** 2021-07-20

**Authors:** Lisandro Ariel Piaggio

**Affiliations:** ^1^Profesor de Urología, Departamento de Ciencias de la Salud, Universidad Nacional del Sur, Bahía Blanca, Argentina; ^2^Professor of Urology, School of Medicine, University of the South, Bahía Blanca, Argentina

**Keywords:** Ricardo Gonzalez, pediatric urology fellowship, professor of urology, sociedad iberoamericana de urología pediátrica, university of buenos aires, academia nacional de medicina de Argentina

Our dear friend Ricardo Gonzalez (Figure 1) passed away on March the 23rd 2021. Ricardo was born in Buenos Aires, Argentina in June 26th 1943 and he graduated from Medical School in 1966 from Universidad de Buenos Aires. He was interested in digestive and biliary surgery and he did a General Surgery Residency in Hospital Militar Central (Buenos Aires). The circumstances of life amalgamate with his personality transformed him into a worldwide famous Pediatric Urologist. The history of how he was pushed to the field of Urology is here…

**Figure 1:**
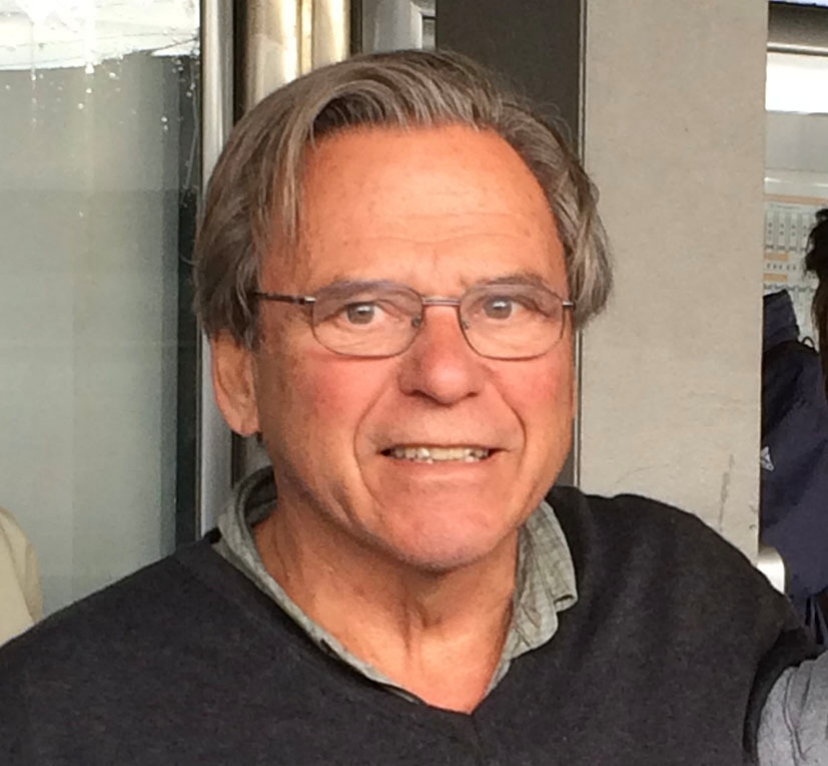
Ricardo González. World Congress of Pediatric Surgery. 2007. Buenos Aires. Argentina.

## The Beginnings

When Ricardo finished his residency program at the age of 25, the Chief of General Surgery Dr. Morera, encourage him to enter the contest for a position in General Urology at his Hospital. Unenthusiastically he applied and won the position, but his thoughts at that time were “I don't like Urology too much…” He felt that he had not good perspectives with the training he received as a general surgeon, and he thought that he would like to do more than “resection of varicose veins, appendix, gall bladder, and, stomach.” Because of this, he sent many letters to academic hospitals around the world and he finally received an offer to travel abroad for additional training. Dr. O.H. Wangensteen, from the University of Minnesota (UM) offered him a position to research in his laboratory. Even though then the idea seemed like a crazy adventure, but he didn't have a second thought and start the journey.

After a year in research in UM, where he published the first use of a laser through a rigid gastroscope to treat bleeding gastric erosions (any similarity to the current urology practice?), he was offered to start his training again as a resident in general surgery in UM. During that period, he met the new chief of the Urology department, Professor F.E.Fraley that –again- tempted Ricardo to join him as a resident in Urology—there is no future in general surgery” he told him. The condition was not only to become a good urologic surgeon but the commitment in research and academic work to eventually become a professor; which he achieve….

## The Way To Pediatric Urology

After becoming a urologist, Ricardo Gonzalez started running the clinics of neurogenic bladder and infertility for some time. When Collin Markland, left the Urology Department at the University, he offered Ricardo to continue his practice in Pediatric Urology. This was a new challenge and he liked very much the idea of taking care of children, but he acknowledges that he did not have enough training in this field. He arranged with the UM and started his training in Pediatric Urology. He traveled to Boston and spent 6 months at Massachusetts General Hospital with Dr. Hardy Hendren and then, for 2 months in Liverpool, UK, to join Herbie Johnston at Alder Hey Hospital for Children. In 1980, he came back to Minnesota starting his full practice in Pediatric Urology and creating the Division of Pediatric Urology. He became an Associate Professor of Urologic Surgery in 1978 and a Full Professor in 1985. He started a Fellowship program and continued a productive academic activity. During the time he spent in Minnesota, Dr. Gonzalez not only trained many residents and fellows but received many foreign doctors from around the world who were eager to rotate with him in his division, and he enthusiasm them in the field.

During his time in Minnesota, he devoted his passion to horse riding and equestrian jumping. He was a very passionate person not only in the field of his profession but with every aspect of life.

## Opening To The World

Many years later, he left Minnesota and moved to Detroit, MC, Miami, FL, and Wilmington, DE in the USA, always running an academic position and training Fellows in Pediatric Urology. He was a professor in Urology at Wayne State University, University of Miami, and Thomas Jefferson University. Since 2006, he was visiting professor at Zurich University, Switzerland, visiting Europe in a scheduled way. When he retired in the USA, he moved to Hannover, Germany, where he continued in academic work as a consultant professor for Medizinsuniversität Berlín Charite and the Children's hospital Auf der Bult in Hannover, Germany, and an International consultant in pediatric urology at the Italian Hospital in Buenos Aires, Argentina.

**Figure 2:**
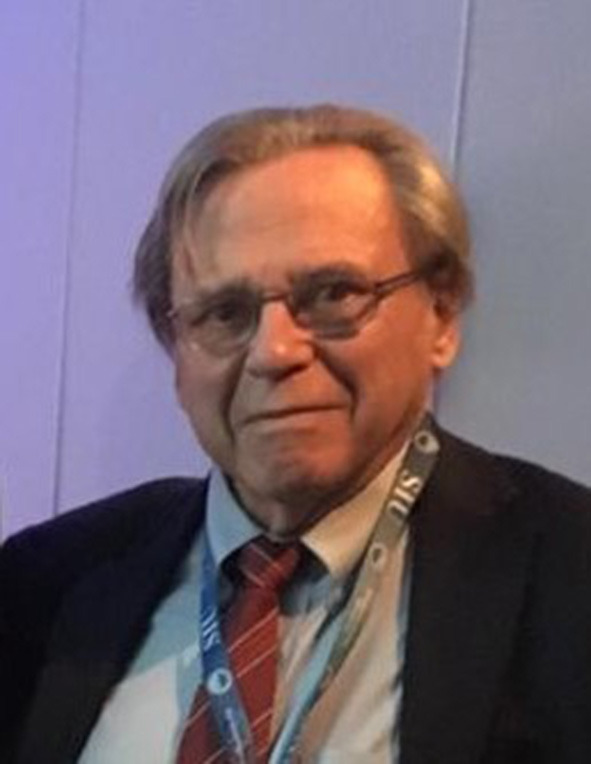
Ricardo González.

**Figure 3:**
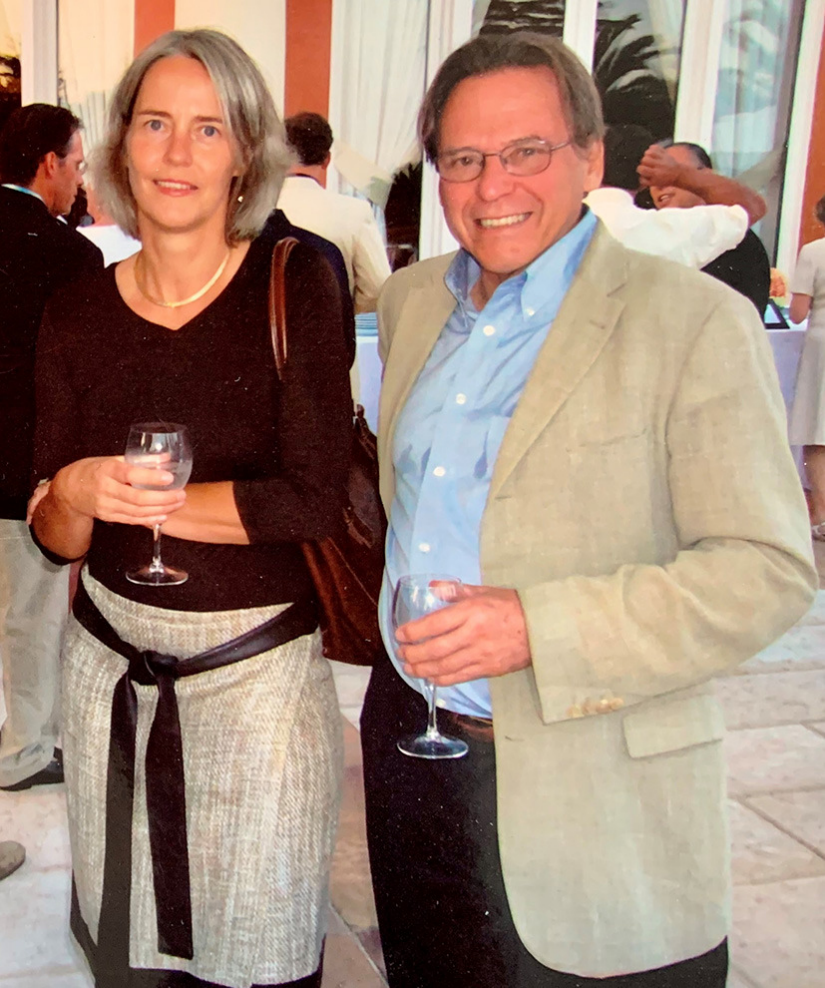
From left to right. Barbara Ludwikowski and Ricardo González. Cocktail in an international meeting.

Dr. Gonzalez was a “Visiting Professor” in 42 Institutions in four continents and was invited for surgical demonstrations in pediatric urology in more than 70 cities in 20 countries. He actively participated in many Scientific Societies being a corresponding or honorary member in at least 31 of them in the USA, Europe, and Latin America. He has been a speaker in many conferences, round-tables, or participated as moderator in scientific meetings on more than 195 occasions (Figures 2, 3) But what he liked the most were the meetings abroad. Ricardo enjoyed traveling, walking outdoors, meeting people and trying typical meals. He was a great talker and he very much like after an exhausting day savoring red wine and good food with friends. He liked more to invite than to be invited, and he would never say “no” unless he had a very good reason. He was very pragmatic, always optimistic, had an excellent mood.

Ricardo Gonzalez was a founding member and first President (1991) of SIUP (Iberoamerican Society of Pediatric Urology). He was very enthusiastic in teaching and training with no barriers and made a bridge merging Pediatric Urologists with a background in Urology or Pediatric Surgery in many countries. Ricardo trained more than 20 fellows in pediatric urology that are working devoted to this field all around the world. Ricardo was a true world ambassador for pediatric urology.

## Academic Achievements

Dr. Gonzalez was recognized around the world as an innovative surgeon whose special interests were urinary incontinence surgery, neuropathic bladder, congenital adrenal hyperplasia, bladder exstrophy, genital reconstruction, and minimally invasive surgery. He received special recognition from the European Society for Pediatric Urology (ESPU), the SIUP, Argentine Pediatric Surgery Society (ACACIP), and the German Society for Pediatric Surgery. He had more than 400 publications including more than 250 peer-reviewed articles and 72 book chapters. He has edited four books, wrote 39 editorials and 24 letters to the editor, 38 invited articles, and eight videos and scientific movies. In June 2016, he was elected as the corresponding member of the National Academy of Medicine in Buenos Aires, Argentina. He has been Specialty Chief Editor for Frontiers in Pediatric Urology since 2013 where he published many papers, edited four Research Topics, and made several editorial contributions. Ricardo has more than 9000 citations and a very high h- index.

## My Personal Experience With Ricardo

I had the privilege to be one of Ricardo's fellows. In some way, I end up training with him by chance. We met for an interview at a dependence of Thomas Jefferson University (PL) where he was at the weekly Fetal Medicine Meeting. “I never miss it” —he told me “I come as a consultant, but I learn a lot.” He was very open-minded and like continuous learning. He was contagious with his interest in training. Then he drove me to Du Pont Hospital for Children where I met the staff and his former fellow in the operating room, July who was performing a laparoscopic pyeloplasty. They were doing their first experience. That *was* Ricardo. In his sixties he was struggling with laparoscopic surgery, to learn and teach a new surgical approach to UPJO, that with open surgery he would do it with his eyes closed. Later on, I made a retrospective review that we presented at the AAP meeting comparing the open and laparoscopic cases. I couldn't find a failure on his hands! Ricardo was an outstanding surgeon; he was studious, tenacious, determined, and sometimes a little obstinate. Later in Europe, he participated in a Robotic Surgery Program at the University of Berlin. He had no fear to face the barriers that new technology would carry.

Despite the pandemic, he wished things to normalize a bit to be able to travel and visit friends. Ricardo was always positive!

When he passed away, a deep sadness touched the heart of many people that knew him around the world. This is what some of them said: teacher of teachers, founder, and pioneer of SIUP, endless investigator and hard worker that enjoyed teaching, great professional and excellent person! Great loss! You leave a legacy to follow. All who knew him will miss Dr. Gonzalez's friendship, leadership, and expertise.

Let me just add, THANK YOU, MASTER! We will miss you a lot!

## Author Contributions

The author confirms being the sole contributor of this work and has approved it for publication.

## Conflict of Interest

The author declares that the research was conducted in the absence of any commercial or financial relationships that could be construed as a potential conflict of interest.

